# The law on compulsory vaccination in Italy: an update 2 years after the introduction

**DOI:** 10.2807/1560-7917.ES.2019.24.26.1900371

**Published:** 2019-06-27

**Authors:** Fortunato D’Ancona, Claudio D’Amario, Francesco Maraglino, Giovanni Rezza, Stefania Iannazzo

**Affiliations:** 1 National Institute of Health, Rome, Italy; 2 Ministry of Health, Rome, Italy

**Keywords:** mandatory vaccinations, Italy, vaccination coverage, National Immunisation Plan

## Abstract

Italy introduced a national law extending the number of compulsory vaccines from four to 10 in July 2017. The implementation placed a further burden on immunisation centres as they were required to cover the increased demand of vaccination by the parents of unvaccinated children. Vaccine coverage (VC) estimated 6 months and 1 year later, at 24 and 30 months (same birth cohort), had increased for all vaccines. At 24 months of age, measles VC increased from 87.3% in 2016 to 91.8% in 2017 and 94.1% at 30 months of age as at June 2018. In six of 21 regions and autonomous provinces, VC for measles was >95%. Despite the implementation of this law, vaccine hesitancy is still a problem in Italy and the political and social debate on mandatory vaccination is ongoing. Regardless of the policy to be adopted in the future, strategies to maintain high vaccination rates and the related herd immunity should be considered, including adequate communication to the population and the implementation of electronic immunisation registries.

## Background

The occurrence of a large measles outbreak in January 2017, triggered the establishment of a new law, adopted in July 2017, which extended the number of mandatory vaccines from four to 10 vaccines for those aged 0–16 years [1]. Vaccinations against pertussis, measles-mumps-rubella (MMR), varicella and *Haemophilus influenzae* type b (Hib) were added to the list of already mandatory vaccines (diphtheria, tetanus, hepatitis B and polio) in the national immunisation plan (NIP). More information on the law was previously published [2]. 

In Italy, individual vaccinations are recorded in the local or regional immunisation information systems (IISs) at the time of vaccine administration. In each of the 21 regions (R)/autonomous provinces (AP), the population for the estimation of VCs is taken from population registers or from healthcare registers. Every year, R/AP send aggregated data to the Ministry of Health (MoH). These data are used to estimate and publish the national VCs for all vaccines included in the NIP for the target age groups (i.e. VC at the age of 24 months, 36 months, 7 years and 16–18 years) [3]. Here, we describe the impact on VC in Italy 2 years after the implementation of the law and the challenges that needed to be overcome in its implementation.

### Vaccination coverage before and after the law

The national VC in Italy from 2013 to 30 June 2018 (1 year after the introduction of the law) can be seen in Table. There was a decline of all VCs since 2014 due to increasing vaccine hesitancy. The impact of the law on the vaccine uptake was positive in the first estimation of all VCs (December 2017) just after 6 months since the implementation of the law [2]. Because evaluating the impact of law was a topic of critical importance to guide a possible revision of the vaccination strategy in Italy, which is currently under discussion in the Italian Parliament, the MoH decided to conduct an extra VC data collection on 30 June 2018 to update the VCs for the birth cohorts already evaluated at the end of 2017.

**Table:** Vaccination coverages by year and vaccine, Italy, 2013–30 June 2018

Vaccine	Number of doses	Year						Difference 2017–18^a^	Range of vaccination coverages among the R/AP in 2018^a^
		2013	2014	2015	2016	2017	2018^a^		
**Vaccination coverage (%) at 24 months^b^**									
Polio	3	95.7	94.7	93.4	93.3	94.6	95.5	+ 0.9	89.4–98.4
Diphtheria	3	95.8	94.7	93.4	93.6	94.6	95.4	+ 0.8	89.4–98.4
Tetanus	3	95.8	94.8	93.6	93.7	94.7	95.5	+ 0.8	89.4–98.4
Pertussis	3	95.7	94.6	93.3	93.6	94.6	95.4	+ 0.8	89.3–98.4
Hepatitis B	3	95.7	94.6	93.2	93.0	94.4	95.2	+ 0.8	88.9–98.3
Hib	3	94.9	94.3	93.0	93.1	94.3	95.0	+ 0.7	88.6–98.4
Measles	1	90.4	86.7	85.3	87.3	91.8	94.1	+ 2.3	82.2–97.5
Mumps	1	90.3	86.7	85.2	87.2	91.8	94.2	+ 2.4	82.2–97.5
Rubella	1	90.3	86.7	85.2	87.2	91.8	94.1	+ 2.3	82.2–97.5
Varicella	1	33.2	36.6	30.7	46.1	45.6	46.7	+ 1.1	4.1–91.7
Meningococcal C	1	77.1	73.9	76.6	80.7	82.6	87.8	+ 5.2	56.9–95.4
Pneumococcal 13v	3	86.9	87.5	88.7	88.4	90.9	92.0	+ 1.1	83.1–96.6
**Vaccination coverage (%) at 36 months^c^**									
Polio	3	96.3	95.7	95.4	94.1	95.1	95.8	+ 0.7	91.4–99.1
Measles	1	92.3	90.7	89.2	88.0	92.4	94.4	+ 2.0	84.5–96.5
**Vaccination coverage (%) in their seventh year of life (plus 6 months)**									
Polio	4	90.9	89.2	87.6	85.7	88.7	92.3	+ 3.6	85.6–96.6
Measles	2	83.5	82.7	83.0	82.2	85.7	90.1	+ 4.4	78.7–94.0

R/AP: regions and autonomous provinces.

^a^ Partial data as at 30 June 2018. Two of 21 R/AP did not send data.

^b^ Vaccination coverage (%) at 30 months for 2018.

^c^ Vaccination coverage (%) at 42 months for 2018.

Source: Italian Ministry of Health.

The data from 2018 show an increase of VCs at the national level (Table) and in almost all the R/AP [4]; at 30 months, VC for MMR vaccine was 94.1% (range 82.2–97.5), with 6 of 21 R/AP having more than 95% children vaccinated (data not shown). For non-mandatory vaccinations (i.e. meningococcal and pneumococcal vaccines) VC were also increasing. However, the data recorded in 2018 showed a wide range in VCs among R/AP, suggesting that there is space for improvement in the implementation of vaccination strategies, especially for vaccinations that were not mandatory before the law.

## Challenges in implementing the new law

### Vaccine offer and delivery

In Italy, vaccination is actively offered to target population groups and administered free of charge by public immunisation services. The Italian health system is decentralised and the NIP is issued by the MoH [5,6], but implemented on a local level by the health authorities in the R/AP according to their regional immunisation plans.

To comply with the requirements of the new law, children aged less than 6 years are required to have complete vaccination cycles to attend educational services and the same applies for students over 6 years of age in order for their parents to avoid being sanctioned with a fine, by the start of the school year in September 2017. After the adoption of the law, the local health units (LHUs), responsible for administering vaccinations to children had a dramatic increase in appointments, both for parent counselling and catch-up vaccinations. The MoH was unable to calculate the exact number of children that would require catch-up vaccinations, but estimated that a total of 4,600,000 doses of the different mandatory vaccines would be needed to cover the full catch-up of the partially vaccinated/not vaccinated from 1 to 16 years of age. 

While some R/AP actively provided planned appointments for the catch-up vaccinations through invitation letters, problems arose when parents did not have a vaccination certificate. In these instances, parents had to contact the LHUs to verify the vaccination status and, eventually, to book an appointment for the vaccination. This resulted in excess requests for public immunisation services and in slowing down their regular activities e.g. administration of other non-mandatory vaccinations (pneumococcal, meningococcal B and C infection, rotavirus and HPV); this slowing down lasted several months. To help alleviate this problem, the MoH permitted all partially/unvaccinated children seeking an appointment for catch up vaccinations at the time of school year opening to have access to the educational services. 

Parental informed consent for vaccinations was used in many LHUs, even if not required by the law; no child was forced to receive any vaccination. In order to identify unvaccinated individuals, the MoH issued a definition of ‘unvaccinated children’ and proposed a table with the catch-up immunisation schedule for children aged up 16 years [7].

### Identification of the unvaccinated children and their catch up

The 2017 measles outbreak was due to low MMR VC among in infants and in adolescents in Italy [4]. In order to identify unvaccinated children aged up to 16 years, in absence of a national IIS, the R/AP used the local or regional IIS [3]. Local immunisation services were supported by educational service managers at schools and preschools, which were required to collect vaccination certificates for all children aged less than 17 years at the moment of school enrolment and transmit the information to LHUs. Difficulties were reported by the educational service managers, due to the different communication strategies to the LHUs in each R/AP. For example, in some schools all the parents had to present the vaccine certificates, while in others the certificates were only requested of children not registered in the local IIS. After the first year following the introduction of the law, all these critical points were gradually solved. 

### Application of penalties

As part of the law, a fine was introduced for parents/guardians refusing vaccination and partially/unvaccinated children under the age of 6 years were not permitted to attend pre-school education services. However, political and social debate, typically fuelled by groups opposed to the law (e.g. ‘free-vax’ movement), led to some R/AP authorities delaying the implementation of the financial fines for unvaccinated children until early 2019, creating inequalities among the R/AP. Self-certification of the vaccine status by the parents was accepted by school managers until March 2019 [8,9]. As the attendance of educational services for children under age of 6 years is on voluntary basis, it was not possible to estimate the number of children to whom access was denied.

### Increasing the population’s knowledge and awareness of the importance of vaccination

In order to raise awareness of the law, the MoH created a website dedicated to vaccinations, with a special section dedicated to the new law [10] and provided a free phone number and two mailboxes dedicated to questions about vaccination that are still active. In addition, five circular letters providing information regarding the new law were sent to public regional and national institutions, health and educational authorities and healthcare professionals all around Italy.

The implementation of the law, resulted in media interest with particular focus on the safety and effectiveness of vaccinations and contributed to increasing the awareness of the importance of vaccination in the population. LHUs, R/AP authorities and scientific societies additionally implemented communication and training activities for public health and healthcare providers. In late 2018, the MoH launched a national TV and internet campaign on the benefits of vaccination using two celebrities as testimonials, a volley ball champion and an astronaut, in order to contrast vaccine hesitancy [11]. The increase of vaccination coverage may be a result of this debate and information campaign raising awareness of the importance of vaccination. A survey conducted by Giambi, and colleagues (Istituto Superiore di Sanità, Rome, Italy) in 2018 (data not shown) compared recent data (following the implementation of the law) with a previous survey conducted in 2016 [12]. They found that the percentage of hesitant parents had decreased in Italy from 15.5% in 2016 to 11.5% (p < 0.001) in 2018 and that the number of anti-vaxxers had decreased from 0.7 to 0.5 (not statistically significant).

## Conclusions

Vaccines have become a national talking point in Italy as a result of the newly introduced law. While reasons for low VC include a low perceived risk regarding vaccine preventable diseases [13], vaccine hesitancy due to low confidence in vaccines, safety concerns and lack of specific recommendations [12]. Prior to the introduction of the new law, attempts to improve the quality of public immunisation services and communication campaigns were not sufficient to have a positive impact on these factors and therefore VC [4,14]. Some of these points have been addressed during the implementation phase of the new law and there are encouraging signals that the situation may have improved as indicated by the survey conducted by Giambi et al. and by the positive trend in VC coverage for the vaccinations that before law were not mandatory, e.g. for measles.

In Italy, mandatory vaccination is still debated and a source of controversy due to unresolved different opinions and the need to strike balance between individual freedom and the public health perspective. After the elections in March 2018, the new government prepared a proposal to revise the law moving towards a more flexible approach in the definition of the mandatory vaccinations, that is now under discussion in the Parliament [15].

There are some limitations that should be considered when interpreting the VC data. The 2018 VC refers to older children (30 months rather than 24 months), which could affect the comparability with the previous year. The absence of data from two R/AP could also have affected the national average and decreased the comparability with 2017 data. The estimation at the end of the first half of 2018 could be less comparable with data collected at the end of the year, due to possible different methods used to estimate numerator and denominator for VCs being the first interannual data collection. The complete 2018 data as at 31 December 2018, were collected and they are currently under validation. The planned implementation of a national IIS may minimise the bias due to the difficulties of local and regional IISs to estimate the number of vaccinated people, given the high mobility in the country, and provide more accurate VC estimates.

Any future change in the law should be accompanied by a strong communication campaign to the population to explain the rationale of such changes and support them with scientific evidence and adequate investments to avoid losing trust in vaccination. The implementation of electronic immunisation registries should be ensured at national level to enforce the monitoring of the vaccination strategy and to rapidly identify areas or population groups with lower coverage.

Whatever the policy to be adopted in the future, strategies to maintain or even improve high vaccination rates and the related herd immunity should be considered. Moreover, with regard to measles, 95% VC among children aged 2 years has been almost achieved, but there are still geographical variations throughout the country. All these aspects should be taken into account when planning effective vaccination strategies.
